# Supplementary material for: Are carbon emissions trading and green financial instruments synergistic? -Comprehensive quantitative research based on content analysis
Source: PLoS One. 2024 Mar 7;19(3):e0298601. doi: 10.1371/journal.pone.0298601 (PMC10919601; doi:10.1371/journal.pone.0298601)
Supplement: S1 Data — (ZIP) [file pone.0298601.s001.zip › Data/地级市Z_F工作报告.zip!/╔╜╢1⁄2/╬1⁄2╖╗╩╨/└┤╫╘╣1⁄2╓┌║┼í╛┬φ┐╦╩2╛▌═°í┐_2018.docx]

2017年全市经济社会发展取得可喜成绩

2017年是新一届市政府履职的第一年。一年来,市政府认真贯彻落实中央和省委、省政府决策部署,在市委坚强领导下,团结带领全市人民,开拓进取,扎实工作,较好地完成了市十七届人大一次会议确定的目标任务。

经济平稳健康运行。预计地区生产总值增长8%左右,社会消费品零售总额增长10.5%左右,进出口总额增长12%左右,固定资产投资完成年度任务。一般公共预算收入539.1亿元,可比增长8.5%。城镇登记失业率控制在2.91%。居民消费价格上涨1.5%左右。省下达约束性指标全面完成。

产业转型加快推进。三次产业结构进一步优化。新发展新型农业经营主体788家,新增“三品一标”认证产品42个。成功举办2017全国农商互联大会,获批创建国家农商互联标准化示范市,成为首批国家农业对外开放合作试验区。规模以上工业经济效益大幅提升。高新技术产业产值占比提高1.03个百分点。7家企业入选国家“两化”融合贯标试点。潍柴集团收入突破2000亿元,荣获中国商标金奖。新登记各类市场主体14.4万户,增长18.7%。规模以上文化企业达到272家。新增4A级物流企业4家、5A级旅游景区1家。全市电商交易额增长26%,快递业务量增长36%。预计旅游消费总额达到770亿元。被确定为国家医养结合示范省先行区。

创新能力持续增强。科技进步对经济增长的贡献率达到58%,全社会研发经费投入占GDP比重达到2.61%。发明专利授权量、国际专利申请量分别居全省第3位和第2位。新增省级以上企业技术中心40家、省级工程实验室6家、院士工作站4家,新组建产业技术创新战略联盟6家。市工业研究院及十大产业创新中心挂牌成立。氢燃料电池项目被列为国家重大创新专项。北大现代农业研究院、北航歌尔机器人与智能制造创新技术研究院、潍坊激光雷达技术研究院等37家高校院所研发机构落户潍坊。雷沃重工被认定为农机行业首个国家级工业设计中心。获批建设中国(潍坊)知识产权保护中心。

改革开放不断深化。“三去一降一补”扎实推进。持续实施降成本“711”专项行动,为企业减负140亿元。重点行业产能利用率稳定在80%以上。新增社会融资规模833亿元。完成规模以上企业规范化公司制改制920家,3家公司在主板上市,新增“新三板”和区域性股权交易市场挂牌企业8家和285家。新设专项投资基金5支,累计实现基金总投入273亿元。全市公共资源交易额达到860亿元。完成事业单位改革任务,39家公立医院、6家科研院所建立法人治理结构。9处国有林场改革顺利完成。承担的48项国家和省改革试点扎实推进。实际到账外资75亿元。进口肉类指定口岸通过国家验收。潍坊机场年客运量达到60万人次。潍坊港年货物吞吐量4200万吨、集装箱35万标箱,国内首条对流铁路冷链班列开通运行。与国外10个城市结为友好城市。风筝会、鲁台会、中日韩产业博览会影响力和承载力进一步增强。

城乡区域统筹发展。全市常住人口城镇化率预计达到60.15%。208个市区重点项目扎实推进,站南广场片区、高铁新片区开发建设加快推进。潍坊综合保税区北区获批设立。4个县市入围全国县域经济百强,2个镇进入全省十强,9个小镇入选省级特色小镇。改造提升农村社区服务中心234个。棚户区改造项目开工8.97万套,完成农村无害化厕所改造20.9万户。重大基础设施加快建设。潍坊港4个5万吨级和2个2万吨级泊位投入运营。潍日高速北段、宝通街东延、潍昌路和央赣路新改建项目建成通车,潍日高速南段、济青高速改扩建工程快速施工。轨道交通启动前期工作。新改建农村公路1568公里。黄水东调一期、引黄入峡后续工程完成建设。农村电网改造升级全面竣工,榆横-潍坊、扎鲁特-青州特高压工程建成投运。

生态环境明显改善。扎实推进生态环境综合整治,集中解决环境突出问题。建立网格化环境监管体系,深入实施“啄木鸟行动”。全市“蓝天白云、繁星闪烁”天数达到277天,PM2.5、PM10、二氧化硫、二氧化氮分别改善9.2%、8.9%、36.1%和5.3%。全面推行河长制,启动“清河行动”,市控以上重点河流COD、氨氮平均浓度分别改善6.1%和9.6%。禁养区畜禽养殖场全部搬迁。新能源公交车占比达到56.2%,成为国家公交都市创建城市。完成造林8.3万亩。节地、节水、节能工作扎实开展。

民生质量稳步提高。民生支出比重保持在80%以上。城乡居民人均可支配收入预计分别增长8%和8.5%。全市剩余1.11万贫困人口、28个省定扶贫工作重点村基本脱贫。新增城镇就业11.2万人,“证卡合一”试点经验在全省推广。职工大病保险制度全面建立,在全省率先实现跨省异地就医直接结算。新改扩建中小学校221所、普惠性幼儿园190所,中小学“大班额”比例全省最低。袁强荣获第44届世界技能大赛金牌。新建全民健身设施1041处。潍水文化生态保护实验区通过国家评估验收。市博物馆创建为国家一级馆。顺利通过全国文明城市、全国卫生城市复审。新建标准化农村社区卫生室227处、城市社区日间照料中心328处、农村幸福院689处。创建为首批国家食品安全示范城市、全国基层中医药工作先进市。荣获巴塞罗那世界智慧城市博览会智慧城市提名奖。“一村(社区)一警务助理”实现全覆盖。安全生产事故起数、死亡人数实现双下降。“破案会战”和“打霸治痞”专项行动成效显著。退役士兵安置和权益保障政策得到有效落实。外事侨务、民族宗教、妇女儿童、防灾减灾、慈善事业、双拥共建、人口计生、老龄、残疾人、审计、统计、海事、海渔、人防、地震、气象、史志、档案等工作取得新进展。

政府自身建设进一步加强。“放管服”改革扎实推进。公布“零跑腿”和“只跑一次”改革事项329项,政务服务事项网办率达到75%,实现“三十一证合一、一照一码”。认真执行市人大及其常委会决议决定,自觉把民主政治协商纳入决策程序,共办理人大代表议案建议152件、政协委员提案359件。编制实施《潍坊市人民政府工作规则》。积极预防和化解行政争议。连续两年位居中国法治政府评估前十名。“12345”政务服务热线完成整合升级。市直部门公用经费压减5%。深入开展“作风建设年”活动。党风廉政建设和反腐败工作扎实推进。

2018年政府工作总体思路和目标任务

今年政府工作总的要求是:全面贯彻党的十九大和中央、全省经济工作会议精神,以习近平新时代中国特色社会主义思想为指导,坚持稳中求进工作总基调,坚持新发展理念,紧扣我国社会主要矛盾变化,按照高质量发展的要求,统筹推进“五位一体”总体布局和协调推进“四个全面”战略布局,牢牢把握走在前列的目标定位,以供给侧结构性改革为主线,加快实施新旧动能转换重大工程,统筹做好稳增长、促改革、调结构、惠民生、防风险各项工作,全面推进“四个城市”建设,坚决打好三大攻坚战,推动质量变革、效率变革、动力变革,实现经济平稳健康发展,民生福祉持续改善。

今年经济社会发展主要预期目标是:地区生产总值增长7%以上,一般公共预算收入增长6.5%,固定资产投资增长8%左右,社会消费品零售总额增长9.5%左右,进出口总额增长8%左右。城镇登记失业率控制在3.8%以内。居民消费价格涨幅3%左右。全面完成省下达的约束性指标。

贯彻落实新发展理念,努力推动高质量发展

要把学习宣传贯彻党的十九大精神作为全年工作的灵魂和主线,坚持“走在前列”的目标定位,贯彻落实新发展理念,进一步深化供给侧结构性改革,坚持以人民为中心的发展思想,狠抓作风建设,努力推动高质量发展,为建设富裕文明充满活力的现代化强市而努力奋斗。

一、全面推进“四个城市”建设

(一)加快建设产业强市。

推进传统产业提质效。深度对接“中国制造2025”,促进全产业链整体跃升。加快“四新”改造。支持企业引入新技术、新产业、新业态、新模式。加快制造业与互联网融合发展。推动集群发展。做大做强行业龙头企业。支持“两群”企业全球布局、配置资源,领办创办特色产业园区和专业配套园区,组建产业联盟,促进产业链垂直整合。强化品牌质量建设。深入开展质量提升行动,加强质量基础设施建设。鼓励企业开展商标国际注册、国际认证,加快培育一批国际知名品牌。增强机遇意识,搞好顶层设计,强化综合整治,坚决打好化工产业安全生产转型升级攻坚战。

促进新兴产业上规模。聚焦聚力新兴产业,强化龙头带动、园区承载、项目支撑、要素保障,加快壮大产业规模。大力发展海洋经济,建设海洋科技产业聚集示范区。抢抓人工智能发展机遇,培育壮大人工智能新兴业态。促进军民融合型产业快速发展。

支持重点服务业快突破。推动生产性服务业向专业化和价值链高端延伸、生活性服务业向精细化和高品质转变。大力发展现代物流业。围绕高铁、公路、港口、机场规划布局物流园区。大力发展现代金融。启动新一轮金融创新发展三年行动计划。大力发展普惠金融、绿色金融。深化投融资改革。大力提升旅游业。高水平编制全域旅游发展规划,打造精品线路,培育壮大市场主体。大力发展总部经济。完善商贸服务设施。优化中心城区商业布局规划。加快发展电子商务。积极发展以医养结合为重点的养老服务业。支持发展健康向上的休闲娱乐业。

放大现代农业新优势。大力推进农业科技创新、模式创新、产业创新、品牌创新,强力推进国家农业开放发展综合试验区建设。实施科技创新工程。实施龙头企业振兴工程。鼓励引导龙头企业延伸产业链条,创新营销模式,带动农业产业转型升级。实施农业品牌引领工程。

(二)加快建设品质城市。

坚持高起点规划。实行“多规合一”,启动新一轮城市总体规划修编。推进建筑容量、界面控制、建筑节能、建筑轮廓线等先进标准落地。打造教育、医疗、养老、商业等功能完备的“15分钟生活服务圈”。

实施高标准建设。严格建筑质量监督管理,实施建筑工程项目竣工联合验收。把海绵城市建设要求落实到每个新建改建项目。大力推进城区雨污分流改造和箱涵溢流整治。推进城区大热源改造。开展城市地下管网普查。打通城市次支路网。分期分批对城市主要道路、重点区域老旧建筑外立面实施改造。规划建设一批口袋公园、便民公厕。实施城市环境提升工程。实施高速出入口和城市主干道两侧绿化提升行动。依托城市周边林场规划建设郊野公园。开展品质小区建设行动。坚持“房子是用来住的、不是用来炒的”定位,加快建立多主体供应、多渠道保障、租购并举的住房制度,促进房地产市场健康发展。

推进高效能管理。深化城市管理体制改革,推进城市管理重心下移。探索推行“路长制”。实施城管网络巡查和第三方监督评测。开展户外广告专项整治。推进智慧交通建设。实施以“城市大脑”为核心的“智慧潍坊3.0”建设。

(三)加快建设活力城市。

提升创新创业水平。围绕产业链部署创新链,围绕创新链配置资源链,进一步发挥科技创新支撑引领作用。提升科技创新平台研发能力和技术攻关水平。实施普惠性创新政策,推进高新技术企业“育苗造林”行动。健全完善技术交易平台。扎实推进国家知识产权强市创建和知识产权保护中心建设。深入推进大众创业、万众创新。创建一批创业型示范镇(街)、示范社区、示范平台。出台人才支撑新旧动能转换工作意见,实施领军型创新创业团队引进培育计划。激发和保护企业家精神,鼓励更多社会主体投身创新创业。大力弘扬劳模精神、工匠精神,建设知识型、技能型、创新型劳动者大军。

深化重点领域改革。深入落实“三去一降一补”重点任务。实施五年资本市场千亿融资计划,完成省定规模以上企业规范化公司制改制任务。支持鼓励上市公司、股改企业增资扩股,提高再融资能力。实施小微企业“双升”战略,推动“个转企”登记。深化财税体制改革。加快农村集体产权制度改革。完善承包地“三权”分置制度,鼓励向新型农业经营主体流转土地,加快实现重点农业区域土地托管服务全覆盖。推进公共采购、用地供应等制度改革。加快国有企业混合所有制改革,推进经营性国有资产统一监管。深化开发区体制机制改革。强化统计执法监督。

推动形成全面开放新格局。全面落实“六六三”开放战略,深入实施“十项计划”,增强招商实效。深入实施外贸转型升级专项行动,加快建设跨境电子商务综合试验区,积极创建自贸协定出口实施示范市。支持企业“走出去”开展国际并购、资产重组、合作研发,深度参与“一带一路”建设,培育本土跨国公司。推进鲁辽大通道、“潍青欧”“鲁新欧”国际多式联运通道健康发展。大力发展会展经济。做好对口支援工作。

(四)加快建设文化名市。

大力发展优秀文化。深化公民思想道德建设和群众性精神文明创建,推进志愿服务制度化。加强文物保护利用和文化遗产保护传承。打造非物质文化遗产项目聚集区,举办中国(潍坊)传统工艺创意设计大赛。完成全国地方志第二轮编纂工作。推进国家历史文化名城、联合国手工艺与民间艺术之都、中国书法名城等品牌创建。鼓励引导民间收藏。组织开展“写潍坊、拍潍坊、演潍坊、画潍坊、唱潍坊”系列活动。

完善提升文化设施。健全现代公共文化服务体系,启动市博物馆群、美术馆群建设。新改扩建10家县级综合档案馆。加快建设镇(街)公共图书馆、文化馆分馆。村(社区)综合性文化服务中心达标率和省定扶贫工作重点村文化大院建成率达到100%。广泛开展群众性文化活动,深入推进文化惠民工程。推进全民阅读,依托社区规划建设便民“城市书吧”。

做强做大文化产业。积极培育文化龙头企业,支持潍坊报业集团、广播影视集团向全媒体转型发展。吸引社会资本投资兴办文化企业,支持文化企业上市或挂牌交易,加快发展多层次文化产品和要素市场。探索建立文化金融服务中心。推进文化产业园区、特色文化小镇建设,打造一批文化产业聚集区和特色文化体验区。办好第三届市民文化节和第二届潍坊文化惠民消费季。提升第八届中国画节、第十一届中国(潍坊)文化艺术展示交易会办会水平。

二、着力抓好四项工作

(一)强力推进项目建设。认真落实市级重大项目建设意见,全面深化“项目建设年”活动。开展集中开工、专项督查、观摩点评等系列活动,建成运营重大项目智慧化管理平台。完善问题直报、分层解决、“3+N”会商解决机制,打通项目快速落地“绿色通道”。

(二)高效推进招商引资。明确招商方向、招商重点,创新招商方式、招商机制。推进专业招商、产业招商。统筹招商力量,实行以业绩为核心的绩效考核。强化产业招商,发挥企业招商主体作用和各类开发区招商主战场作用。积极创新招商模式。推行风险共担、利益共享的“招商合伙人”制度。组织“千企千商”走访、“潍商回归”和重点区域集中招商活动。坚持人才集聚。把人才资源作为战略资源、第一资源,优化人才结构,做好“育用引”三篇文章。改善营商环境。加快建立与国际通行营商环境体系相衔接的指标体系。

(三)有序推进棚改旧改。坚持“阳光征收、不与民争利、群众工作法”三项原则,完善棚改旧改、房屋征收政策。坚持整体拆迁、片区开发。按照商品房的质量标准优先建设群众安置房。稳步推进老旧小区改造提升,完善配套设施。

(四)全面推进重大基础设施建设。加快潍莱高铁及配套枢纽工程建设,推动京沪高铁二通道开展前期工作,济青高铁建成通车。完成潍日高速南段主体工程,济青高速改扩建主体工程完成90%以上。加快潍坊港5万吨级航道和码头建设。有序推进机场搬迁。轨道交通加快报批,力争年内开工建设。实施潍县路升级改造工程,开工建设东外环、南外环、南绕城高速。利用两年时间,打通北宫东街、文化路、东方路等21条城区道路,有序推进志远路、友爱路、向阳路等上跨下穿青银高速、胶济铁路通道建设。加快原油超市项目建设。推进山东-河北特高压环网工程、临朐抽水蓄能电站、滨海大型LNG调峰储配站建设,加快充电站(桩)配套设施建设。积极推动峡山水库胶东调蓄水源地工程,加快黄水东调二期建设,抓好重点水源联通工程。加强信息、物流等基础设施网络建设。

三、坚决打好三大攻坚战

(一)防范化解重大风险。加强金融风险防控。加大银行业信用风险处置化解力度。扎实做好不良担保圈、担保链“破圈解链”工作。强化金融风险应急处置。持续抓好安全生产。官梯深入开展“安全生产责任落实年”活动。健全网格化监管体系,实行安全监管“实名制”。抓好隐患排查和专项整治。实现全市交通运行状态实时监控。着力维护社会稳定。创建国家防震减灾示范城市,筹建市救灾物资储备中心。全面加强应急管理工作。深化“平安潍坊”建设,加快市县镇村四级综治中心平台建设。

(二)高质量完成脱贫任务。坚持精准扶贫、精准脱贫,确保年内实现“全部兜底脱贫”目标。注重扶贫与扶智、扶志相结合。实施贫困村整体提升工程,有序推进省定扶贫工作重点村建设美丽乡村。探索创新“互联网+”社会扶贫模式,完善社会扶贫供需对接机制。实施最严格的监督执纪,以“零容忍”态度严惩扶贫领域腐败。

(三)强化生态环境治理。进一步加大生态环境整治力度,按要求做好中央环保督察反馈问题整改。打赢蓝天保卫战。全面落实《潍坊市大气污染防治条例》,持续开展生态环境综合治理。推进能源结构调整,完成煤炭减量替代任务。持续抓好散煤清洁化治理。健全完善企业环保预警、倒计时整改机制。推进出租车绿色改造。提升水污染防治水平。全面深化河长制。有序推进市县镇三级污水处理设施改造提升。扎实开展潍河、弥河等9个河道综合整治工程。严格划定农村饮用水水源保护区,实施白浪河水库水源地保护工程。加强土壤污染管控和修复。建立土壤环境基础数据库。完善病死畜禽无害化处理体系。推广土壤熏蒸修复、精准施肥和绿色防控技术。抓好耕地质量提升和减肥增效示范县创建。推进固废危废处置项目建设。加大生态保护力度。加快建立生态补偿机制。加快南部山区森林生态屏障建设。强化北部沿海滩涂湿地和海域生态保护修复。建成市县两级智慧环保平台。开展全民节水行动,推广“零增地”发展模式,加快建设节约型社会。

四、统筹区域协调发展

(一)构建现代化城镇体系。在更高层次上深度谋划,有序推进青潍一体化；以“三带引领、两河开发”为统领,统筹推进全域一体化；以推进交通基础设施互联互通为重点,加快推进“七城”一体化；树立大市区理念,把市区作为一个整体规划建设管理。中心城区优先发展总部经济、楼宇经济、城市综合体、生产性服务业、高端服务业,有选择地发展先进制造业、科研科创孵化基地以及大健康、文化休闲等现代服务业。坚持产城融合,加快建设宜业宜居宜游的站南广场片区、高铁新片区。支持高新区发展光电产业、VR产业、动力产业、生命健康产业,打造区域国际化先行区。支持滨海区发展高端装备制造、海洋精细化工、高端石化、生物医药产业,打造产城一体的产业集聚区。强化综合保税区对外开放服务功能,打造开放发展服务高地。放大峡山区生态优势,打造独具特色的生态发展示范区。

(二)加快推进县域发展。坚持“多予少取放活”原则,推进县域经济突出特色发展。加强顶层设计和资源统筹,支持县域发展壮大先进制造业为主体的实体经济。调整优化市以下财政体制,完善财政转移支付制度,加大市级产业发展引导基金对县域产业发展支持力度。完善县域科学发展考核机制。鼓励支持镇域竞相发展,打造好1个国家级和9个省级特色小镇,培育一批市级特色小镇。

(三)实施乡村振兴战略。坚持农业农村优先发展,加快编制实施乡村振兴战略规划。聚焦产业兴旺。实施质量兴农战略,推进农业由增产导向转向提质导向。加快农村一二三产业融合、农业内部产业融合。完善“龙头企业+合作社+家庭农场”等股权利益联结机制。增强抗旱防汛能力,实施“藏粮于地、藏粮于技”战略,严守耕地红线,确保粮食安全。聚焦生态宜居。开展农村人居环境三年整治行动,大力改善路水电气暖讯房等基础设施。开展河道、坑塘治理,提升绿化亮化美化水平。加快推进农村危房改造,有序推进改厨工作。坚持不懈推进“厕所革命”。提高集中供水质量。推进“四好农村路”建设。保护好乡村特色风貌和田园风光。完成农村社区服务中心建设提升三年任务。聚焦乡风文明。加强农村公共文化建设。开展移风易俗行动。探索开展“乡贤回归”工程。聚焦治理有效。创新农村社会治理,健全自治、法治、德治相结合的乡村治理体系。加强农村法治建设,推进平安村庄建设。完成村(社区)“两委”换届选举工作。聚焦生活富裕。重塑城乡关系,坚持以工补农、以城带乡,建立健全城乡融合发展体制机制和政策体系。鼓励工商资本、社会资本参与乡村振兴。完善社会化服务体系,推进农户与现代农业有机衔接。设立乡村振兴基金。建立新型农民培训制度。大力发展农村集体资产合作社、土地合作社、农村劳动力合作社,拓宽农民增收渠道。

五、提高民生保障和社会治理水平

今年民生支出比重保持在80%以上,可量化项目保障标准全部达到或超过省均水平。

(一)优先发展教育事业。实施第三期学前教育行动计划。启动义务教育优质均衡县创建,高质量完成破解中小学“大班额”任务,开展中小学生课外负担综合治理,加快推进普通高中综合改革。优化职业院校办学结构。鼓励企业依法参与举办职业教育、高等教育。支持潍坊医学院创建潍坊医科大学,加快推进山东海洋技术大学筹建工作。落实好第二期特殊教育提升计划。启动建设北辰现代教育示范区。创建国家级现代教育综合改革试验区。

(二)实现更高质量更充分就业。注重解决结构性就业矛盾,推动全日制、集中式就业向灵活式、分散式就业拓展,鼓励通过新兴业态扩大就业。落实企业工资指导线和工资集体协商制度,构建和谐劳动关系。

(三)完善社会保障体系。落实好被征地农民社会保障政策,完善机关事业单位养老保险制度改革配套措施,健全大病保险、异地就医、职工长期护理保险、门诊特殊慢性病等医保制度,继续推进民生综合保险。开展残疾人精准康复服务。完善综合社会救助体系。

(四)推进健康潍坊建设。完成医疗机构设置规划和空间布局规划,完善分级诊疗服务体系。加快重点医疗机构集团化发展和医联体建设,提升乡镇卫生院医疗服务水平。推进家庭医生签约服务。启动市妇女儿童健康中心建设。实施“幸福呼吸”和“关爱新生命”公益项目。推进人口计生工作“三个转变”,科学应对“全面两孩”生育高峰。落实好公共场所母婴设施配置要求。实施“振兴中医”行动计划。启动实施重大传染病疫情分级分类处置。完善养老服务体系。持续开展“食安潍坊”品牌引领行动,大力推进餐饮质量安全提升工程。加强药品质量安全监管,落实药品供应保障制度。积极推进国家农产品质量安全城市创建。广泛开展全民健身运动。

(五)加强社会治理。落实“谁执法谁普法”责任制,推动全民尊法学法守法用法。加快推进公共法律服务体系建设。进一步畅通信访渠道。深入推进社会信用体系和企业产品与服务质量信誉体系建设。强化市级公共信用信息平台数据归集、共享应用和信用监管。大力支持国防建设。

努力建设人民满意的服务型政府

牢固树立以人民为中心的发展思想,把人民群众的小事当作自己的大事,从人民群众关心的事情做起,从让人民群众满意的事情做起,扎实推进高效政府、责任政府、法治政府、廉洁政府建设。

不断提升政府效能。深化“放管服”改革,提高行政审批效率。加强部门协作配合、融合合作,汇聚抓落实强大合力。加快“互联网+政务服务”建设,深入推进“多证合一、一照一码”改革,扩大“证照分离”试点。完成政务信息系统整合共享工作。着力打造以“12345”热线为主渠道的综合性为民服务平台。

更加突出专业能力。提升公务员专业素养和专业能力。完善以专业知识和专业能力为导向的考核评价机制。立足岗位需求,定期组织开展专业培训和更新知识培训。探索实行公务员聘任制。

大力弘扬务实作风。深入落实中央八项规定实施细则精神。扎实开展“不忘初心、牢记使命”主题教育。持续开展“作风建设年”活动。大力推行“一线工作法”。落实容错纠错机制,鼓励干部干事创业。

扎实推进依法行政。深入落实法治政府建设实施纲要,把政府工作全面纳入法治轨道。自觉接受人大及其常委会的法律监督、政协民主监督、媒体舆论监督,强化审计监督。切实做好人大代表议案建议和政协委员提案办理工作。严格执行地方性法规和政府规章制定程序,继续加强重点领域立法。落实重大行政决策法定程序,强化合法性审查。全面深化政务公开。完善政府法律顾问制度,严格规范行政执法行为。发挥行政复议、行政应诉作用,妥善化解行政争议。

全面加强廉政建设。严格落实全面从严治党主体责任,认真履行“一岗双责”。坚持把纪律和规矩挺在前面。强化对权力运行的制约和监督。严格规范招投标活动和政府采购管理,规范公共资源配置交易,严管民生资金、民生项目。深入推进反腐败斗争,保证干部清正、政府清廉、政治清明。
